# Dysregulated erythropoietin-producing hepatocellular receptor A2 (EphA2) is involved in tubal pregnancy via regulating cell adhesion of the Fallopian tube epithelial cells

**DOI:** 10.1186/s12958-018-0403-5

**Published:** 2018-09-03

**Authors:** Huan Jiang, Xiao-Yi Yang, Wei-Jie Zhu

**Affiliations:** 1 Department of Reproductive Endocrinology, Longgang District Maternal and Child Healthcare Hospital, 6# Ailong Road, Longgang Central District, Shenzhen City, 518172 People’s Republic of China; 2 Institute of Reproductive Immunology, College of Life Science and Technology, Jinan University, 601# Huangpu Da Dao Xi, Guangzhou City, 510632 People’s Republic of China

**Keywords:** Receptor, EphA2, Cell adhesion, Fallopian tube, Pregnancy, Tubal

## Abstract

**Background:**

Tyrosine kinase receptor erythropoietin-producing hepatocellular receptor A2 (EphA2) is abundant in the endometrium and plays a role in the establishment of eutopic implantation. A similar molecular mechanism may exist between uterine implantation and tubal implantation, therefore EphA2 involvement in tubal pregnancy is suspected. Due to the limited availability of human Fallopian tube specimens, EphA2 expression in human Fallopian tube epithelium remains largely unknown.

**Methods:**

A total of 31 women with tubal pregnancy and 41 non-pregnant women with benign uterine diseases were enrolled in this study. Immunohistochemistry was used to investigate the expression pattern of EphA2 in the Fallopian tube epithelium of non-pregnant women (*n* = 29) and women with tubal pregnancy (*n* = 17). The changes of EphA2 and its activated form, phosphorylated-EphA2 (Pho-EphA2), in the Fallopian tube epithelium from non-pregnant women (*n* = 12) and women with tubal pregnancy (*n* = 14) were compared by quantitative RT-PCR and western blot assay.

**Results:**

EphA2 was expressed throughout the Fallopian tube epithelium, including the isthmus, the ampulla and the infundibulum. EphA2 concentration remained unchanged throughout the whole menstrual cycle, irrespective of menstrual phases and tubal regions. EphA2 mRNA in the Fallopian tube epithelium did not differ between normal women and women with tubal pregnancy (*P* > 0.05). With respect to the protein level, a significantly higher ratio of EphA2 over Pho-EphA2 was shown in women with tubal pregnancy (*P* < 0.05).

**Conclusions:**

EphA2 is widely expressed in human Fallopian tube epithelium in a temporospatial-independent manner. Dysregulated EphA2 and its phosphorylation-dependent regulatory mechanism may unexpectedly enhance the cell adhesion activity of the Fallopian tube epithelial cells, leading to a mis-contact between the Fallopian tube epithelium and the embryo.

## Background

An ectopic pregnancy is a pathological event characterized by embryo implantation occurring outside the uterine cavity. Fallopian tube localization accounts for roughly 98% of all human ectopic pregnancies [1]. Tubal damage as a result of surgery or infection (especially Chlamydia trachomatis), smoking and in vitro fertilization are the dominant risk factors for tubal ectopic pregnancies [1]. However, tubal pregnancy is now thought to be a consequence of molecular dysregulation [1]. One major reason underlying this phenomenon is that adhesion molecules such as integrins and mucin 1, etc., which mediate blastocyst adhesion to the uterine wall, are also expressed in the Fallopian tube epithelium [2]. Unexpected molecular changes may afford an opportunity for the undue remain of embryo in the Fallopian tube [2, 3]. Cell adhesion molecules are abundant in human epithelial cells and crucial for intercellular adhesion. It is presumed that cell adhesion activity of the Fallopian tube epithelial cells is under the fine control of cell adhesion molecules and that changes in their concentrations can alter the cell adhesion of the Fallopian tube epithelium, leading to false recognition between mother and fetus [4–6]. Therefore, it is rational to propose that cell adhesion molecules expressed in the Fallopian tube epithelium may actively participate in the pathogenesis of tubal pregnancy.

Erythropoietin-producing hepatocellular receptor A2 (EphA2) is a member of the largest known tyrosine kinase receptor family, which is well known to generate multiple cellular responses including cell adhesion and migration [7–10]. Activation of EphA2 is phosphorylation-dependent. Binding of EphA2 to its predominant EphrinA1 ligand enables its phosphorylation (Pho-EphA2) [11, 12]. This leads to the initiation of a downstream signaling cascade, which results in the internalization and degradation of Pho-EphA2 itself [11, 12]. It is found that EphA2 is highly localized in endometrial epithelial cells accompanied by its Ephrin ligand expressed on embryonic trophoblasts, and EphA2 is suggested to mediate the establishment of uterine implantation by regulating embryo-maternal contact [13, 14]. Furthermore, EphA2 can promote the invasion and proliferation of the human extravillous trophoblastic cells probably via regulating the ephrin-A1 ligand [15]. As a similar molecular mode may exist between uterine implantation and tubal implantation, it is reasonable to expect EphA2 involvement in tubal pregnancy. However, EphA2 expression in human Fallopian tube epithelium has not been fully documented and the role of EphA2 in tubal pregnancy remains unknown as yet.

We have previously demonstrated in vitro study that EphrinA1 could induce EphA2 activation in human fallopian tubal epithelial cells, accompanied by the up-regulation of Pho-EphA2 [16]. Moreover, we used fibronectin-coated culture plates to interact with the cultured Fallopian tube epithelial cells and found that EphA2 activation could attenuate its cell adhesion activity, which, when disrupted, may be associated with certain pathological events occurred in the Fallopian tube, such as tubal pregnancy [16]. However, the expression pattern of EphA2 in human Fallopian tube epithelium remains presently unknown. Therefore, the present study was undertaken to investigate the EphA2 expression in human Fallopian tube epithelium by immunohistochemical analysis. Considering the involvement of EphA2 activation in regulating cell adhesion activity of the Fallopian tube epithelial cells, the changes of EphA2 and its activated form, Pho-EphA2, in the Fallopian tube epithelium were further compared between non-pregnant women and women with tubal pregnancy using quantitative reverse transcription-polymerase chain reaction (qRT-PCR) and western blot assay, aiming to elucidate the roles of EphA2 and its phosphorylation-dependent mechanism in the molecular pathogenesis of tubal pregnancy.

## Methods

### Specimen collection of human fallopian tubes

Thirty-one women who had been diagnosed as tubal pregnancy and undergone surgeries in the First Affiliated Hospital of Jinan University, PR China were collected, and 41 non-pregnant women who had undergone hysterectomies for benign uterine diseases were set as controls. Informed consent was attained from each patient and ethical approval for this study was obtained from the Local Research Ethics Committee. All the enrolled subjects should meet the following criteria: (1) a normal history of fertility and regular menses; (2) a definitive date of the last menstrual period; and (3) no use of exogenous hormonal drugs or intrauterine devices in the 6 months preceding the surgery. Women with tubal pregnancy who were determined to have morphological abnormalities in the Fallopian tubes or benign uterine diseases such as uterine leiomyoma, adenomyosis and endometriosis after surgical operations were excluded.

Among all the enrolled subjects, the Fallopian tube samples from 17 tubal pregnant women (mean age 27.7 ± 3.7 years; range 26–34 years; gestational weeks 8.5 ± 0.9) and 29 non-pregnant women (control) (mean age 37.6 ± 3.8 years; range 35–45 years) were paraffin embedded and used for immunohistochemistry. Each Fallopian tube sample with tubal pregnancy was further divided into 2 portions, implantation site (*n* = 17) and non-implantation site (*n* = 17), according to the distance apart from the gestational sac. The implantation site was defined as the circumference originating 5 mm from the gestation sac, while the non-implantation site was 10 mm outside the gestation sac [17]. The controls were further divided into 4 groups based on the corresponding endometrial morphology [18]: early-stage of the proliferative phase (days 1–5; *n* = 8), mid- and late-stages of the proliferative phase (days 7–14; *n* = 6), early-stage of the secretory phase (days 15–18; *n* = 7) and mid- and late-stages of the secretory phase (days 19–28; *n* = 8). All of the paraffin blocks were cut and prepared for immunohistochemical staining.

Fresh Fallopian tube samples obtained from the other 14 tubal pregnant women (mean age 28.9 ± 4.1 years; range from 28 to 37 years; gestational weeks 8.9 ± 0.9 weeks) and 12 non-pregnant women (mean age 40.3 ± 4.2 years; range from 35 to 46 years) were used for qRT-PCR and western blot analysis. Each Fallopian tube tissue with tubal pregnancy was similarly classified into implantation site and non-implantation site. Samples used for mRNA analysis were immersed into Trizol solution, and all fresh specimens were frozen at − 70 °C until analysis.

### Immunohistochemistry

The paraffin-embedded materials were cut into 4-μm-thick sections. These sections were deparaffinized and boiled in citrate buffer solution for 15 min in an oven. After washing with phosphate-buffered saline (PBS), the sections were immersed into 3% hydrogen peroxide for 10 min to quench endogenous peroxidase activity. Afterwards, mouse anti-human EphA2 monoclonal antibody (1:200; ab118882; Abcam, Cambridge, UK) was applied overnight at 4 °C according to the EnVision™ system (Zhongshan Goldenbridge Biotechnology Co., Ltd., Beijing, China). The sections were washed in PBS and incubated for 30 min with horse radish peroxidase (HRP)-conjugated goat anti-mouse IgG (BM2101; Dako Cytomatin, Glostrup, Denmark). Diaminobenzidine (DAB) substrate-chromogen system (Zhongshan Goldenbridge Biotechnology Co., Ltd., Beijing, China) was used as the color-developing substrate. The slides were applied in two 5-min incubations and then counter-stained with hematoxylin. Normal gastric mucosa with known presence of EphA2 served as a positive control. The primary antibody was replaced with non-immune mouse serum IgG to serve as a negative control. All slides were viewed under an E200 microscope (Nikon, Tokyo, Japan). Sections were quantified over ten randomly selected fields of view. Positive unit (PU) assessed by the QW550 image analysis system (Leica, Wetzlar, German) was used to describe the EphA2 expression [19, 20]. The formula was PU = 100 (Ga - Gb)/Gmax. Ga and Gb were the average gray scale of positive staining and background, respectively. Gmax was the maximum gray scale of image analytical system.

### Quantitative RT-PCR

Total RNA was extracted from the fresh Fallopian tube samples using Trizol total RNA extraction reagent (Takara, Dalian, China). Agarose gel electrophoresis was used to ascertain the quality of the RNA products by the presence of 28S rRNA, 18S rRNA and 5S rRNA bands, with a 2:1 ratio of 28S rRNA to 18S rRNA. cDNA was reverse transcribed from the extracted RNA using RT kit (Takara, Dalian, China). The primers used for EphA2 were 5’-AAGACCCTGGCTGACTTT-3′ (forward) and 5’-GTTCACCTGGTCCTTGAGT-3′ (reverse), while the primers for 18sRNA were 5’-CCTGGATACCGCAGCTAGGA-3′ (forward) and 5’-CCTGGATACCGCAGCTAGGA-3′ (reverse). Real time RT-PCR was carried out using an ABI 7300 real-time PCR system (ABI, Carlsbad, USA) with 5 μl diluted cDNA and 0.5 μl primers. The programmed thermocycler included 40 cycles of predenaturation at 95 °C for 10 min, followed by denaturation at 95 °C for 15 s, annealing at 60 °C for 15 s, and extension at 72 °C for 30 s. All reactions were performed in triplicate. Relative quantification of EphA2 mRNA was calculated using the comparative Ct method (ΔΔCt = ΔCt _EphA2_− ΔCt_18sRNA_).

### Western blot

The total protein content extracted from the fresh Fallopian tube samples was resolved by sodium dodecylsulfate-polyacrylamide gel electrophoresis (SDS-PAGE), after which the proteins were transferred to nitrocellulose membranes. The membranes were blocked for 2 h in 10% bovine serum albumin (BSA) (DingguoChangsheng Biotechnology Co., Ltd., Beijing, China) at room temperature, followed by incubation at 4 °C overnight with the primary antibodies, rabbit anti-human polyclonal antibodies against EphA2 and Pho-EphA2 (1:1000; 6997S and 6347S; Cell signaling technology Co., Ltd., Boston, USA) and mouse anti-human monoclonal antibody against glyceraldehyde phosphate dehydrogenase (GAPDH) (1:1000; sc-59,540; Santa Cruz, California, USA). After washing, the membranes were then incubated for 1 h with the appropriate HRP-conjugated secondary antibodies, goat anti-rabbit IgG (1:800; A0208; Beyotime Biotechnology Co., Ltd., Shanghai, China) or rabbit anti-mouse IgG (1:1000; D031402; Dako Cytomatin, Glostrup, Denmark). Protein bands were identified using ECL (Pierce, Rockford, USA). The bands were semi-quantified using Bio-Rad Quantity One software. EphA2 was normalized to GADPH, and Pho-EphA2 was normalized to EphA2.

### Statistical analysis

All statistical analyses were carried out using SPSS V.14. (SPSS, Chicago, USA). Differences among groups were determined by one-way analysis of variance (ANOVA) followed by Student–Newman–Keuls (SNK) multiple-comparison test. Results were presented as mean ± standard deviation (SD). *P* values < 0.05 were considered statistically significant.

## Results

### EphA2 expressions in human fallopian tube epithelia of non-pregnant women

**Fig. 1:**
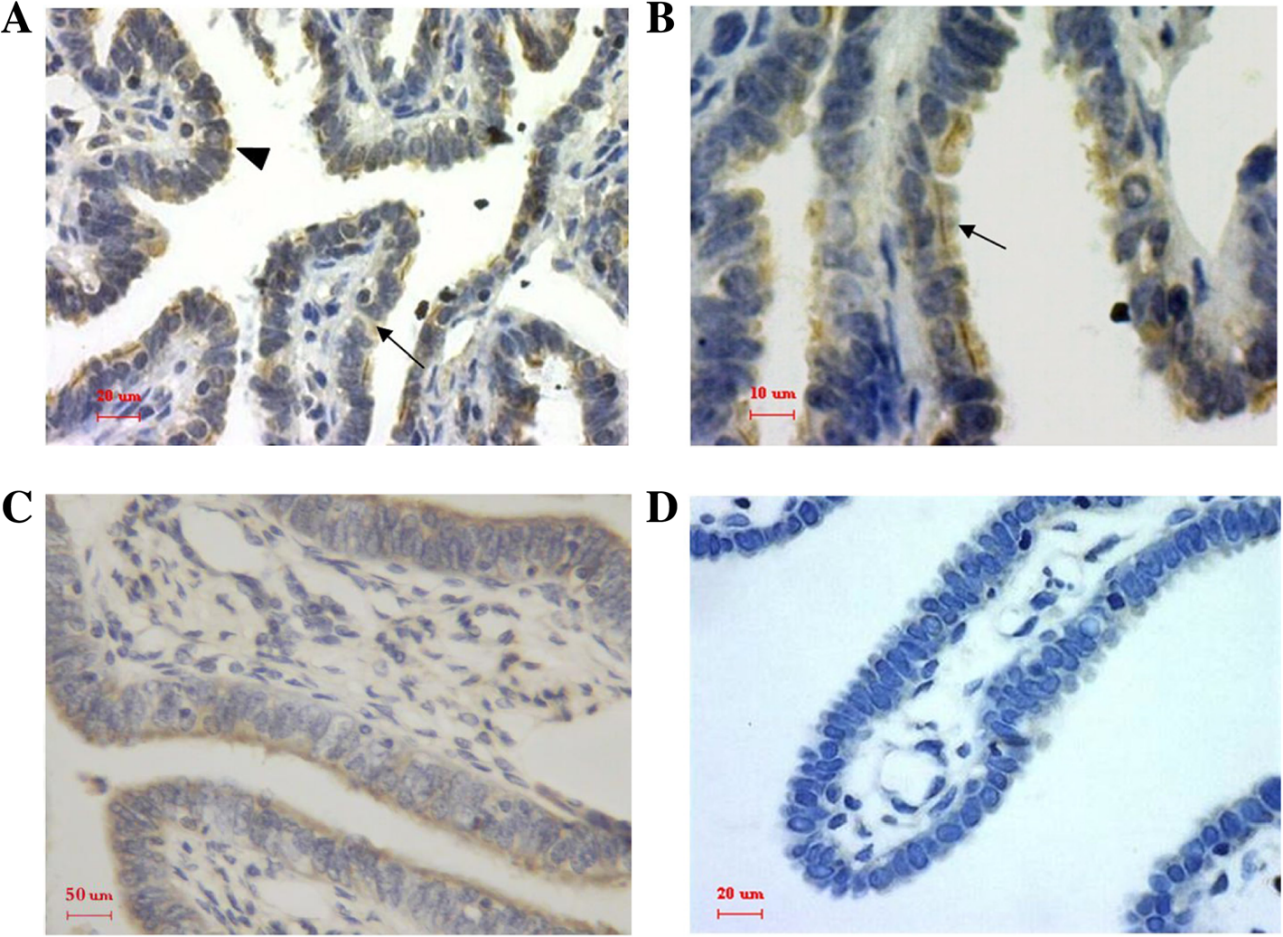
EphA2 immunostaining (brown) on the Fallopian tube epithelium during normal menstrual cycle. **a** EphA2 was expressed on the apical membrane (triangle) and in the cytoplasm (arrow) of the tubal epithelial cells, including the ciliated cells and the secretory cells, while the staining intensities in the cytoplasm were weaker than those on the apical membranes. There was no staining evident in the interstitial cells. **b** EphA2 was present in the cilia of the ciliated cells (arrow). **c** Stronger stains were shown on the apical membranes of the tubal epithelial cells. **d** Negative control

Immunohistochemistry confirmed that EphA2 was present throughout the Fallopian tube epithelium, embracing the isthmus, the ampulla and the infundibulum. EphA2 showed stronger staining on the apical membrane of both the ciliated cells and the secretory cells as well as the cilia. The cytoplasm of the ciliated cells and the secretory cells showed positive staining of EphA2, but the staining intensities in the cytoplasm were weaker than those on the apical membranes (Fig. 1a and b). No staining was detected in the interstitial cells. No significant differences of the staining intensities could be found among different sites of the Fallopian tube during the same menstrual phase or among different menstrual phases at the same tubal region (*P* > 0.05) (Table 1).

**Table 1:** EhpA2 expressions in human Fallopian tube epithelia

Menstrual cycle phases	Exact menstrual cycle days	N	The Fallopian tube sites		
			Isthmus	Ampulla	infundibulum
Early-stage of proliferative phase	Days 1–5	8	4.57 ± 2.38	6.15 ± 2.23	5.16 ± 2.47
Mid- and late-stages of proliferative phase	Days 7–14	6	6.43 ± 3.02	7.02 ± 3.15	6.29 ± 2.36
Early-stage of secretive phase	Days 15–18	7	6.57 ± 3.29	6.54 ± 3.07	6.21 ± 2.98
Mid- and late-stages of secretive phase	Days 19–28	8	5.43 ± 2.59	5.91 ± 2.54	5.73 ± 2.65

Positive unit (PU) values were determined by immunohistochemistry technique, and data were presented as mean ± standard deviation (SD). *P* > 0.05, compared between the two groups in the same line

### Changes of EphA2 and pho-EphA2 in human fallopian tube epithelia of women with tubal pregnancy

**Fig. 2:**
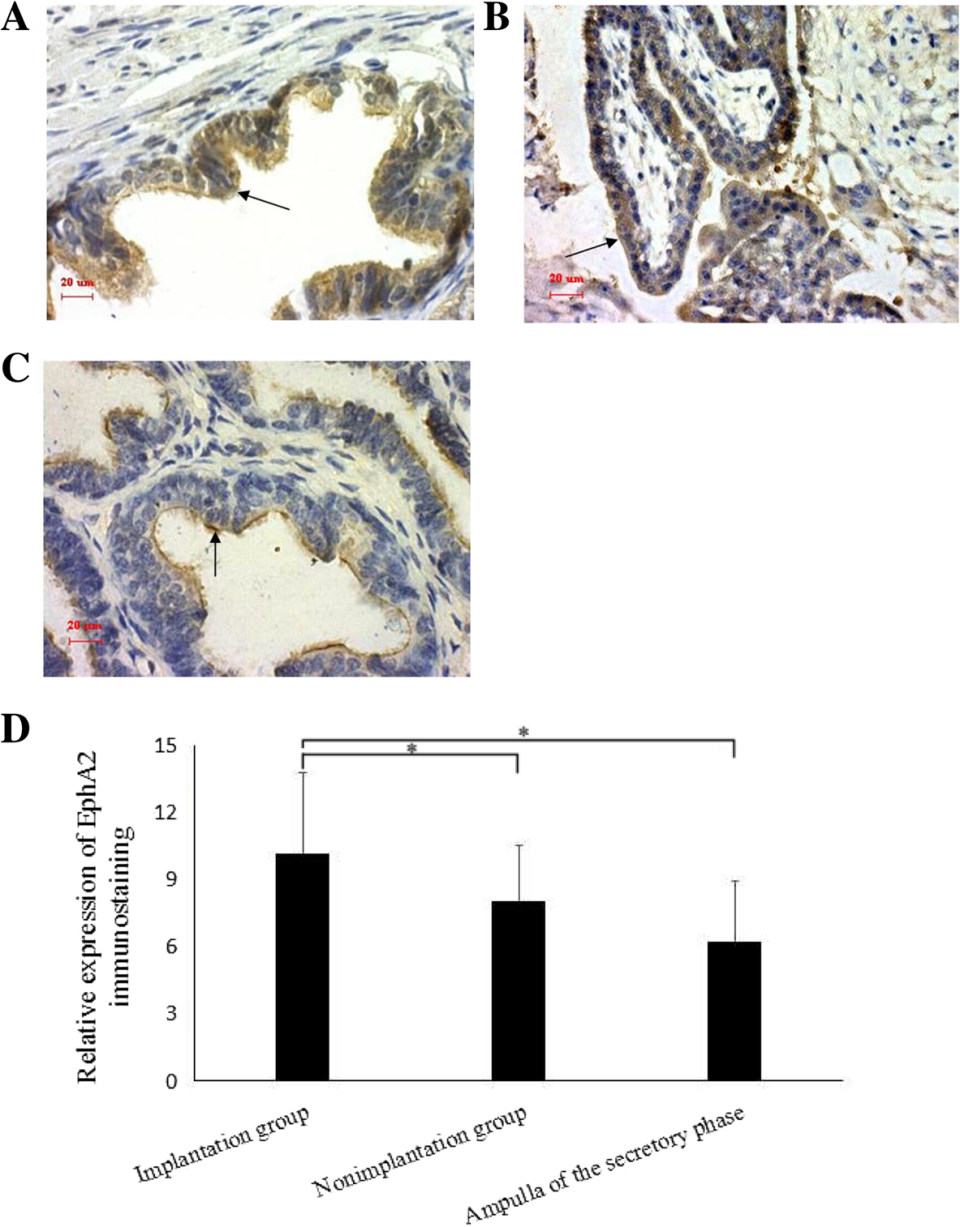
EphA2 immunostaining (brown) on the Fallopian tube epithelium during tubal pregnancy. **a** EphA2 was present in the epithelial cells located in the implantation site of tubal pregnancy (arrow). **b** EphA2 was present in trophoblast cells found in the implantation site (arrow). **c** EphA2 was present in the non-implantation site of tubal pregnancy, and a stronger staining was evident on the cytomembrane (arrow) than that in the cytoplasm. **d** EphA2 expressions on the Fallopian tube epithelia with tubal pregnancy (*n* = 17) and the secretory Fallopian tube epithelia of normal controls (*n* = 15). EphA2 expression in the implantation group presented with the highest-level, compared with that in the non-implantation group and the control group (*P* < 0.05). EphA2 expressions in the latter two groups did not differ (*P* > 0.05)

Immunohistochemical staining confirmed the presence of EphA2 in the Fallopian tube epithelia of women with tubal pregnancy, including the implantation site and the non-implantation site (Fig. 2a, b and c). The strongest staining of EphA2 was noted in the implantation group (10.16 ± 3.59), compared with that in the non-implantation group (8.03 ± 2.49) and the control group (6.20 ± 2.72) (*P* < 0.05). With regard to the latter two groups, the EphA2 staining intensities were similar (*P* > 0.05) (Fig. 2d).

**Fig. 3:**
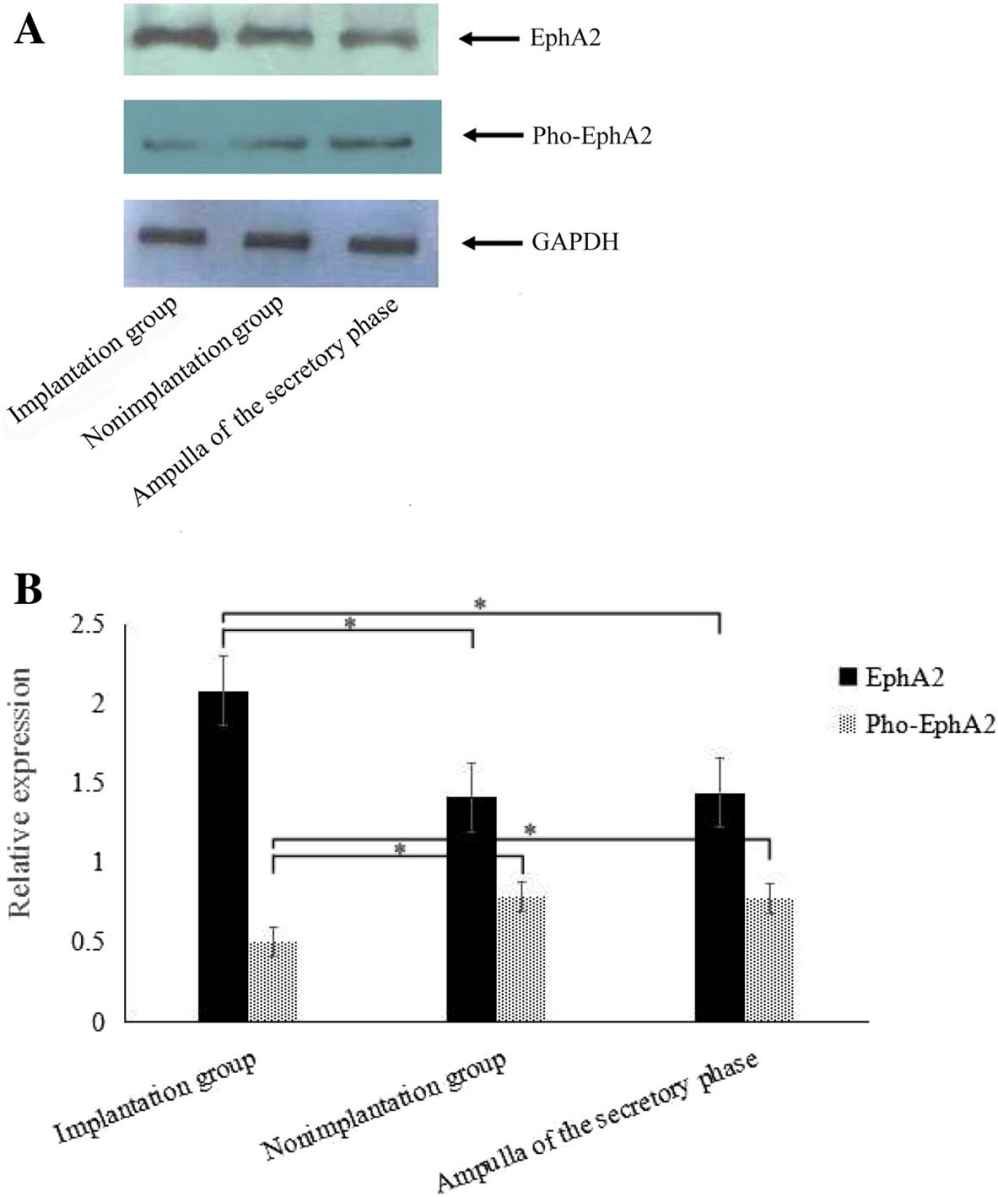
Comparisons of EphA2 and Pho-EphA2 protein expression between the samples derived from the normal Fallopian tubes of the secretory phase (control) (*n* = 12) and the tubal pregnant tissues, which were further divided into the implantation group (*n* = 14) and the non-implantation group (*n* = 14). **a** Western blot showed positive bands of EphA2 and Pho-EphA2 in both the implantation and non-implantation groups of tubal pregnancy. **b** The highest EphA2 accompanied by the lowest Pho-EphA2 was detected in the implantation group, compared with that in the non-implantation group and the control group (*P* < 0.05). EphA2 and Pho-EphA2 protein expressions in samples of the latter two groups were similar (*P* > 0.05)

Western blot analysis revealed positive bands of EphA2 and Pho-EphA2 in both the implantation group and the non-implantation group (Fig. 3a). Similar to the results obtained from immunohistochemical assessment, the implantation group had a significantly increased level of EphA2 (2.08 ± 0.87) compared to that in the non-implantation group (1.41 ± 0.64) and the control group (1.44 ± 0.62) (*P* < 0.05). As for Pho-EphA2, a remarkably decreased level was seen in the implantation group (0.51 ± 0.25) compared to that in the non-implantation group (0.79 ± 0.37) and the control group (0.78 ± 0.35) (*P* < 0.05). However, both EphA2 and Pho-EphA2 levels did not differ between the non-implantation group and the control group (*P* > 0.05) (Fig. 3b).

### EphA2 mRNA in human fallopian tube epithelia between non-pregnant women and women with tubal pregnancy

**Fig. 4:**
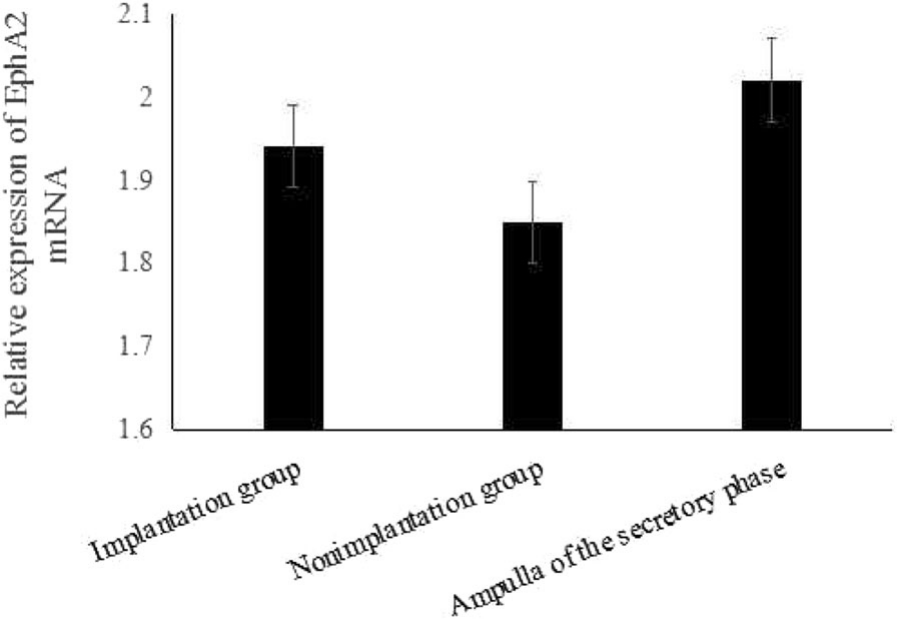
EphA2 mRNA levels did not differ among the control group (*n* = 12), the implantation group (*n* = 14) and the non-implantation group (*n* = 14) (*P* > 0.05)

The EphA2 mRNA levels were similar among the implantation site, the non-implantation site and the Fallopian tube from non-pregnant women (control), with the corresponding values referring to 1.94 ± 0.51, 1.85 ± 0.74 and 2.02 ± 0.55, respectively (*P* > 0.05) (Fig. 4).

## Discussion

To the best of our knowledge, this is the first study to compare the expressions of EphA2 in human Fallopian tube epithelia between non-pregnant women and women with tubal pregnancy. Acted like the endometrium, the morphology of the Fallopian tube epithelium could similarly respond to a menstrual cycle [21, 22]. Therefore, in the present study, the normal Fallopian tube samples were categorized according to the corresponding endometrial morphology, and EphA2 was revealed to widely express in human Fallopian tube epithelium in a temporospatial-independent manner. An increased EphA2 accompanied by a decreased Pho-EphA2 was noted in the Fallopian tube epithelium with tubal pregnancy. EphA2 activation is autophosphorylation-dependent and the elevated Pho-EphA2, induced by EphA2 activation, could attenuate the cell adhesion activity of human Fallopian tube epithelial cells [16]. Combined with the present data, it is speculated that the aberrant EphA2 in the Fallopian tube epithelium with tubal pregnancy, as indicated by a decreased ratio of Pho-EphA2 over EphA2, may unexpectedly enhance cell adhesion activity the of Fallopian tube epithelial cells, leading to a mis-contact between the Fallopian tube epithelium and the embryo.

Investigation of pathological events occurring within the Fallopian tube necessitates considerations of the tubal microenvironment, of which the homeostasis is largely regulated by the molecules expressed on the Fallopian tube epithelium. Tubal pregnancy is currently accepted to be a direct consequence of molecular causes, considering that molecules capable of mediating cell adhesion have been confirmed to play a critical role in the pathogenesis of tubal pregnancy [23]. Eph receptor tyrosine kinases as well as their ligands, ephrins, are important regulators in cell-to-cell interaction and have been proven to play crucial roles in cell migration and adhesion during embryonic development in mammals [24]. EphA2 is abundant in human endometrial epithelial cells and its ligand ephrin is expressed in the blastocysts. A previous study revealed that the Eph-ephrin A system could regulate the initial embryo-maternal contact during the cross-talk period that preceded embryo implantation in the endometrium [14]. Similar regulatory mechanism may exist in the process of tubal implantation. However, evidence pertaining to the role of EphA2 in tubal pregnancy is scarce. The present study revealed that EphA2 was presented in both the ciliated and secretory cells of the Fallopian tube epithelia, and a temporospatial-independent pattern of EphA2 expression was noted. The levels of EphA2 in the Fallopian tube epithelia remained unchanged throughout the whole menstrual cycle, irrespective of menstrual phase or Fallopian tube region. The morphology and physiology of the Fallopian tube epithelium, including the ciliated and secretory cells, can be regulated by sex hormones fluctuating with the menstrual cycle [21]. Such a temporospatial-independent pattern may, in a sense, imply that sex hormones have no impact on EphA2 expression in the Fallopian tube epithelium.

It is well-recognized that overexpression of EphA2 is merely presented in malignant cells, but in non-transformed epithelial cells, EphA2 displays a relatively low level of expression [25–27]. Similar to this pattern, EphA2 was noted to be less abundant in the normal Fallopian tube epithelial cells in contrast to a significant increase in those from women with tubal pregnancy. One possible explanation for this phenomenon may be that EphA2 and its regulatory mechanism are initiated improperly under certain pathological conditions. Moreover, EphA2 is reported to not only function as a marker in transformed cells, but also possess the property to aggravate the malignant progression. Overexpression of EphA2 in non-transformed mammary epithelial cells is sufficient to promote a malignant phenotype [27]. Thus, we tended to speculate that the overexpression of EphA2 presented in the Fallopian tube epithelium may represent its active participation in tubal pregnancy via increasing the cell adhesion activity of the Fallopian tube epithelial cells but not only a simple result caused by tubal implantation.

An increased level of EphA2 was shown in the implantation group compared with that in the non-implantation group and the control group by western blot. However, it seemed a paradox that we failed to find any discrepancy in EphA2 mRNA in the Fallopian tube samples among these three groups. Previous studies concerning the linkage between EphA2 and malignant tumors indicated that an elevated EphA2 found in malignant tumors was a result of deficient phosphorylation of EphA2 itself, leading to the consequent blockage of EphA2 degradation. In other words, insufficient Pho-EphA2 could accordingly give rise to an excessive storage of EphA2 [28, 29]. This conclusion may shed some light on the present data. The presence of increased EphA2 was accompanied by a decrease in Pho-EphA2, implying that the homeostasis of EphA2 in the Fallopian tube epithelium was hampered during tubal pregnancy, and the increased level of EphA2 may occur on the basis of insufficient phosphorylation of EphA2 itself. The activation of EphA2 is coupled with its autophosphorylation, characterized by an elevated ratio of Pho-EphA2 over EphA2, which could attenuate the cell adhesion activity of the Fallopian tube epithelial cells [16]. In the present study, a decreased ratio of Pho-EphA2 over EphA2 was detected in the implantation site of the Fallopian tube epithelium. We hypothesized that this may disclose an inactivated state of EphA2, which would on the contrary unexpectedly enhance the cell adhesion activity of the Fallopian tube epithelium. Thereafter, a false recognition between fetus and mother may be evoked to take place within the specific site of the Fallopian tube epithelium.

Ciliary motion is closely associated with female fecundity by regulating the processes of ovum pickup and transport [30]. Altered activity of cilia was reported to play a pivotal role in the pathogenesis of tubal pregnancy, based on the fact that women presenting with decreased cilia consequently suffered from tubal pregnancy [31]. EphA2/Ephrin-A1 signaling could attenuate cell adhesion activity of the Fallopian tube epithelial cells [16]. EphA2 was shown in the cilia of the tubal ciliated cells, suggesting a possibility that EphA2 may have some implications in the modulation of ciliary activity by regulating cell adhesion. However, the precise mechanisms about how the inactivated EphA2, characterized in the present study as an increased ratio of EphA2 over Pho-EphA2, influenced the ciliary motion still need to be further elucidated.

There are still certain limitations in the present study we need to take into consideration. Firstly, there were a limited number of samples included in this study which may weaken the strength of our evidence. Secondly, there were some age differences between non-pregnant women and women with tubal pregnancy since it is difficult to obtain Fallopian tube specimens from normal young non-pregnant women for ethical reasons. Lastly, Fallopian tube samples from women with tubal pregnancy can only be collected after an implantation event has occurred, making it difficult to directly identify the causal linkage between EphA2 and tubal pregnancy.

Many efforts have been made to clarify the mechanisms responsible for EphA2 activation during pathological events, yet there is still largely unexplored [32–34]. EphA2 activation can suppress tumor cell growth and hence EphA2 has been established as a therapeutic target for malignant tumors by hindering the progression of tumor invasion [35]. Known to be one of the key molecules responsible for regulating cell adhesion activity, it is plausible to assume that EphA2 activation and its downstream signaling could also be established as a molecular target in medical therapy for tubal pregnancy by decreasing cell adhesion activity of the Fallopian tube epithelial cells to avoid the mis-recognition at the maternal-fetal interface.

## Conclusions

In conclusion, we have demonstrated that EphA2 is expressed in human Fallopian tube epithelium in a temporospatial-independent manner. EphA2 and its phosphorylation-dependent regulatory mechanism seem to be activated only under pathological conditions. The presence of a reduced Pho-EphA2 accompanied by an elevated EphA2, a state that may be recognized as EphA2 inactivation, maybe involved in the molecular pathogenesis of tubal pregnancy through up-regulating cell adhesion activity of the Fallopian tube epithelial cells. A better understanding of EphA2 and its signal pathways is of significance to illustrate the molecular pathogenesis of tubal pregnancy. Further investigations with larger samples are necessary to complement our present study.

## Abbreviations

BSA: Bovine serum albumin
DAB: Diaminobenzidine
EphA2: Erythropoietin-producing hepatocellular receptor A2
GAPDH: Glyceraldehyde phosphate dehydrogenase
HRP: Horse radish peroxidase
PBS: Phosphate-buffered saline
Pho-EphA2: Phosphorylated EphA2
PU: Positive unit
qRT-PCR: Quantitative reverse transcription-polymerase chain reaction
SDS-PAGE: Sodium dodecylsulfate-polyacrylamide gel electrophoresis
